# Advanced Imaging Modalities in the Detection of Cerebral Vasospasm

**DOI:** 10.1155/2013/415960

**Published:** 2013-02-06

**Authors:** Jena N. Mills, Vivek Mehta, Jonathan Russin, Arun P. Amar, Anandh Rajamohan, William J. Mack

**Affiliations:** ^1^Department of Neurosurgery, University of Southern California, 1200 North State Street, Suite 3300, Los Angeles, CA 90033, USA; ^2^Department of Radiology, University of Southern California, 1200 North State Street, Suite 3300, Los Angeles, CA 90033, USA

## Abstract

The pathophysiology of cerebral vasospasm following aneurysmal subarachnoid hemorrhage (SAH) is complex and is not entirely understood. Mechanistic insights have been gained through advances in the capabilities of diagnostic imaging. Core techniques have focused on the assessment of vessel caliber, tissue metabolism, and/or regional perfusion parameters. Advances in imaging have provided clinicians with a multifaceted approach to assist in the detection of cerebral vasospasm and the diagnosis of delayed ischemic neurologic deficits (DIND). However, a single test or algorithm with broad efficacy remains elusive. This paper examines both anatomical and physiological imaging modalities applicable to post-SAH vasospasm and offers a historical background. We consider cerebral blood flow velocities measured by Transcranial Doppler Ultrasonography (TCD). Structural imaging techniques, including catheter-based Digital Subtraction Angiography (DSA), CT Angiography (CTA), and MR Angiography (MRA), are reviewed. We examine physiologic assessment by PET, HMPAO SPECT, ^133^Xe Clearance, Xenon-Enhanced CT (Xe/CT), Perfusion CT (PCT), and Diffusion-Weighted/MR Perfusion Imaging. Comparative advantages and limitations are discussed.

## 1. Introduction and Historical Perspective

Cerebral vasospasm is a delayed complication of subarachnoid hemorrhage. It generally occurs 4–14 days after aneurysmal rupture and is associated with morbidity and mortality rates between 10 and 30% [1]. While changes are commonly observed in the large caliber conveyance arteries, effects on the smaller vessels of the microcirculation, including alterations in blood brain barrier permeability, may be equally important in determining clinical impact. Such factors may account for the higher incidence of vasospasm as defined by imaging criteria (“radiographic vasospasm”) than rates of neurological dysfunction (“clinical vasospasm”).

Early diagnosis and treatment could potentially prevent and/or minimize delayed ischemic neurological deficits (DIND). Advances in clinical management of cerebral vasospasm have lagged far behind innovations in brain imaging. Given that radiographic vasospasm does not strongly associate with DIND, efforts have refocused on understanding pathophysiology through advanced correlative imaging. Technological developments have enabled accurate assessment of tissue oxygenation, metabolic uptake, and cerebral perfusion. This paper will review imaging modalities applied to the detection of cerebral vasospasm and the diagnosis of DIND. A historical background is presented, and fundamental applications are considered.

**Figure 1:**
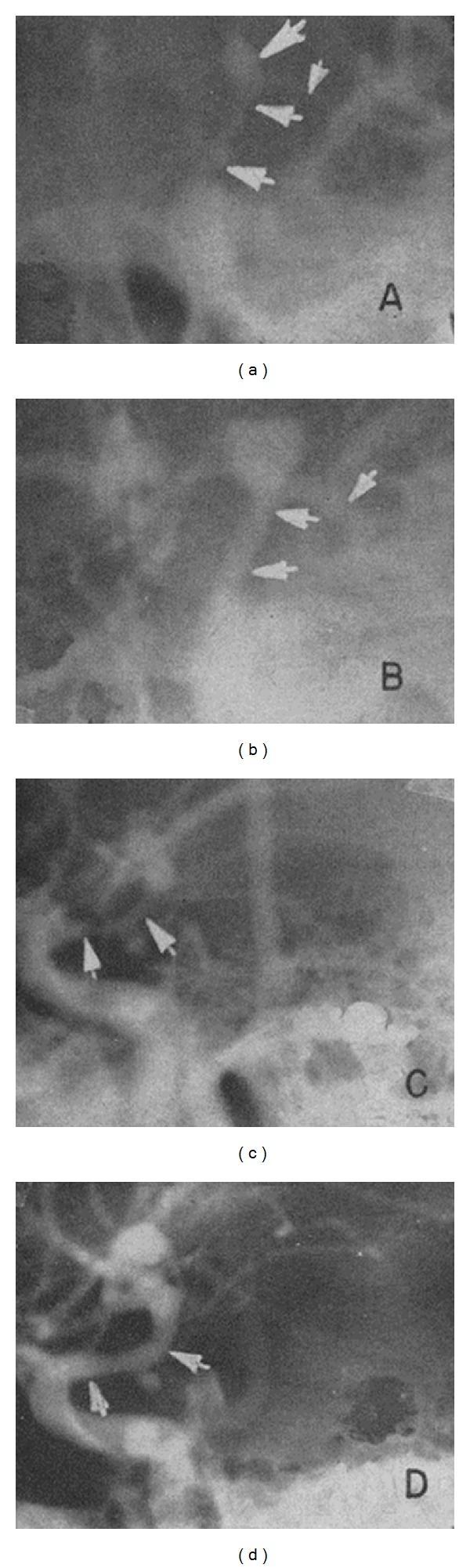
First radiographic demonstration of cerebral vasospasm at bifurcation of left ICA using arteriography adapted from [2].

Radiographic evidence of vasospasm secondary to aneurysmal rupture was initially documented in 1951 (Figure 1) [2], more than two decades after the advent of cerebral angiography [3]. Between 1972 and 1982, efforts focused on developing effective alternatives to invasive cerebral angiography for evaluation of vasospasm. During this time, physiologic modalities were introduced, including ^133^Xe Clearance, Positron Emission Tomography (PET), and Xenon-Enhanced CT (Xe/CT), respectively. Rather than providing anatomical visualization of target vessels, these techniques examined regional cerebral blood flow (CBF) and characterized metabolic parameters.

Further advances occurred in the early 1980s. Digital Subtraction Angiography (DSA), with higher resolution imaging capabilities, replaced cut-film angiography and rapidly became the gold standard for radiographic documentation of cerebral vasospasm. Transcranial Doppler Ultrasonography (TCD) was developed as a noninvasive technique for detecting blood flow velocities of intracranial vessels. This revolutionized the clinical management of vasospasm by providing noninvasive monitoring in the weeks following subarachnoid hemorrhage. DSA and TCD remain the standards for comparative analysis in the assessment of novel imaging modalities. Magnetic Resonance Angiography (MRA) and CT Angiography (CTA) are anatomical/structural imaging techniques incorporated into clinical practice in the mid to late 1990s. Lower risk and rapidity of acquisition compared to DSA have rendered these studies practical for first line vessel imaging in most facilities. 

In the past 25 years, advanced perfusion imaging modalities have been developed. These include HMPAO SPECT (1988), Perfusion CT (PCT) (2001), and combined Diffusion-Weighted and MR Perfusion Imaging. Direct examination of cerebral blood flow is coupled with computational assessment of regional perfusion. Coregistration allows potential differentiation of marginally viable penumbral tissue from dense ischemic core. 

The following sections of this paper serve as a basic review of available imaging modalities applicable to the diagnosis of cerebral vasospasm. Each considers the conceptual basis of a particular technique and underscores the applications and potential limitations.

## 2. Transcranial Doppler Ultrasonography (TCD)

Transcranial Doppler (TCD) Ultrasonography was first introduced in the early 1980s as a noninvasive method for the detection of blood flow velocities in the middle, anterior, and posterior cerebral arteries (MCA, ACA, and PCA) [4]. Many studies have since examined technique and clinical utility. Serial measurements provide critical hemodynamic information that requires relatively little postprocessing [5]. This technique utilizes a hand-held microprocessor-controlled Doppler transducer that directly contacts the cranial skin overlying an acoustic (or insonation) window, an area where the cranial bone is thin or a naturally occurring foramen/fissure is present. The transtemporal window is most commonly utilized to evaluate flow velocities in the MCA, PCA, and ACA. Other insonation windows include transorbital for evaluation of the ophthalmic arteries and cavernous segments of the ICA and transforaminal for evaluation of the posterior circulation. 

A relatively low frequency (2 MHz) pulse-waved ultrasonic signal is emitted that can penetrate the cranial bone, interrogate the intracranial vessels, and transmit data regarding cerebral blood flow velocities. Ultrasonographic technology and data analysis are based on the Doppler Effect principle. The TCD probe emits a wave of a given frequency (*f*_*o*_) and detects the altered echo frequency (*f*_*e*_) of the wave that emanates back. The difference between these two frequencies (*f*_*d*_) is the Doppler shift. Using this principle, the velocity of a moving object can be calculated according to the following equation (*c* is the speed of the emitted wave and *Θ* is the angle between the wave and the direction of movement):

(1) $$\begin{array}{c} V=\frac{c\times f_{d}}{2\times f_{o}\times \cos\Theta }. \end{array}$$

**Figure 2:**
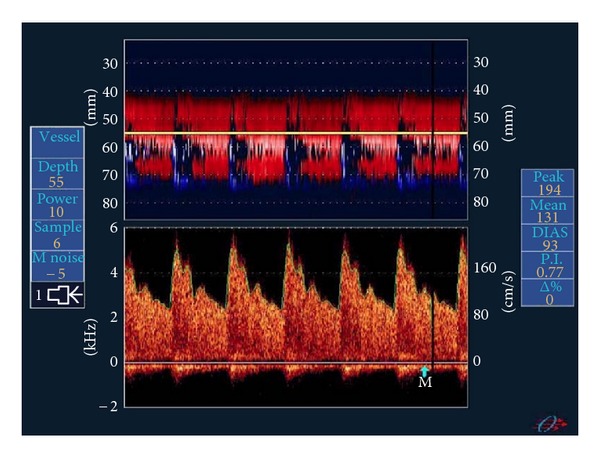
TCD. Doppler velocities demonstrating moderate MCA vasospasm using M mode (above) and pulse-wave Dopper (below). This figure is reproduced from [7].

Interpretation of TCD velocities is based upon the inverse relationship between vessel lumen area and blood flow velocity according to the Continuity Equation [6] (Figure 2). However, normal values can be influenced by both physiological factors and patient characteristics. Flow velocities can vary naturally from minute to minute. Recordings are inherently dependent upon vascular anatomy, angle of insonation, vessel diameter, cerebral blood flow (CBF), and collateral circulation. Confounding factors include mean arterial blood pressure (MAP), PCO_2_, intracranial pressure, age, sex, pregnancy, and arousal state [7]. Pathological conditions including edema or increased intracranial pressure can fundamentally affect the speed, waveform, or turbulence of cerebral blood flow [8–10]. For clinical relevance, TCD velocities must be interpreted in proper context.

Differentiating elevated TCD velocities secondary to vasospasm versus other causes is critical. This can be accomplished by measuring velocities in the cervical internal carotid artery (ICA) in addition to the intracerebral vessels and creating ratios for clinical use. TCD velocities have been used to create several clinically relevant predictive indices including the Lindegaard Ratio (ratio of MCA TCD velocity to ipsilateral ICA TCD velocity) [11], the Jakobsen Spasm Index (ratio of TCD velocity to CBF via ^133^Xe) [12], the Vasospasm Probability Index (VPI) [13], and the Ipsilateral/Contralateral MCA mBFV. The Lindegaard Ratio is especially useful for vasospasm diagnosis. A *V*_MCA_/*V*_ICA_ ratio >3 is suggestive of cerebral vasospasm. A similar velocity ratio profile between the basilar and extracranial vertebral arteries can be used to assess for basilar vasospasm [14]. Such ratios aim to overcome the confounding effects of systemic hemodynamic factors such as changes in cardiac output or blood pressure (e.g., during “triple H” therapy of hypervolemia, hypertension, and hemodilution).

Due to lack of radiation risk, relatively low cost and ease/rapidity of bedside administration, TCD measurements are practical for serially monitoring the progression of cerebral vasospasm. However, attempts to correlate TCD velocities with angiographic studies and neurological outcome measures have generated varied results [15–18]. The technique is limited by a lack of high-resolution anatomic detail. Further, its accuracy and utility are highly operator-dependent. Patient movement, aberrant vessel course, aneurysm clip artifacts, and suboptimal insonation windows can inhibit the detection of pathological velocities [10]. While TCD measurements demonstrate relatively high specificity, diagnostic sensitivity remains controversial. A 2001 meta-analysis reported that, for MCA vasospasm, overall TCD specificity was 99% and sensitivity 67%, with a PPV of 97% and NPV of 78%. For the ACA, ICA, posterior circulation, and examinations of the distal cerebral microvasculature, TCD values were significantly less sensitive and specific [16, 19–22]. A consensus statement in 2004 underscored these results, supporting the conclusion that TCD is a reliable predictor for the absence of angiographic vasospasm (high negative predictive value) at flow velocities < 120 cm/s and for the presence of angiographic vasospasm (high positive predictive value) at flow velocities > 200 cm/s in the MCA territory [9, 17, 19]. Velocity measurements between 120 cm/s and 200 cm/s may require further technical maneuvers (manual carotid compression [23]) or additional clinical information to improve diagnostic accuracy. Moreover, intermediate TCD values generally do not reliably correlate with angiographic vasospasm [17]. Clinically, trends in TCD values or interval changes from prior readings are often more informative than absolute values.

Several newer TCD techniques reduce operator dependence. Power M-mode (PMD) TCD is a technique that facilitates localization of the acoustic window and enables simultaneous visualization of multiple vessels. Currently, the most widely utilized technique for bedside vasospasm detection, PMD-TCD provides information on blood flow velocity, direction, and depth [6]. Transcranial Colour-Coded Duplex Sonography (TCCS) allows for a two-dimensional representation of the basal arteries and incorporates color-coded signals to indicate directionality. This technique provides more accurate parameter measurements and reproducibility, as the entire course of the cerebral vessels can be adequately visualized [6].

## 3. Digital Subtraction Angiography (DSA)

**Figure 3:**
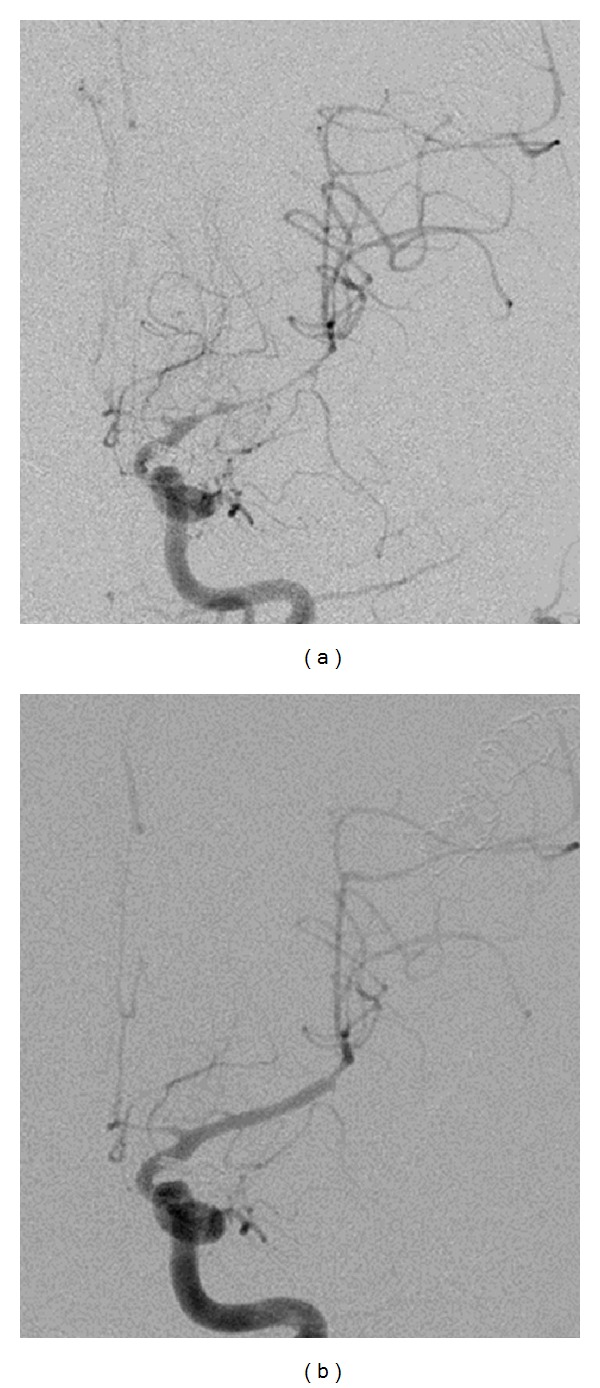
Digital Subtraction Angiography. Severe vasospasm of the left middle cerebral artery demonstrated on digital subtraction angiography (a). Postangioplasty angiogram demonstrates normal caliber of the native vessel (b).

Digital Subtraction Angiography (DSA) was first introduced in 1980 as a direct method of vessel caliber assessment. The technique was designed to improve previous angiographic methods and generate high contrast, high resolution, low artifact images using digital subtraction [24] (Figure 3). DSA is a fluoroscopic examination. Images are acquired through time-controlled X-rays synchronized to the injection of an iodinated contrast medium into the vasculature via a catheter. Images are produced by digitally subtracting extraneous aspects of the image so that the vessels can be visually isolated and evaluated. “Digital subtraction” involves recording a precontrast image, often called a “mask,” and subsequently removing the mask from later images.

Today, direct visualization by DSA remains the gold standard for radiographic diagnosis of cerebral vasospasm and is often performed when clinical vasospasm is suspected. The severity of vasospasm is classified by vessel caliber. Subjective grading schemes have been proposed. Vasospasm has been regarded as severe if vessel caliber reduction is >50%, moderate if between 25–50%, and mild if reduction is <25%, with respect to two-dimensional diameter [25]. DSA is sensitive and specific for detection of proximal vessel narrowing and allows for potential simultaneous endovascular interventions such as balloon angioplasty and/or selective intra-arterial delivery of vasodilatory medication. In addition to vessel caliber, other parameters that can be quantified by DSA include transit time and the interval between different phases of the circulation.

Three-dimensional rotational angiography allows for the acquisition and reconstruction of vessel images. Processing permits more detailed and informative visualization with fewer acquired sequences. Studies have reported nearly 100% sensitivity and specificity for DSA (two-dimensional) confirmed vasospasm. However, operators have also found 3D reconstructions less informative than standard, two-dimensional DSA due to the presence of “pseudo spastic” vessel segments [26]. As with other 3D imaging techniques, elements of the raw data can be lost in computational reconstruction and processing as algorithms are often designed to produce smooth and aesthetically appealing images. Software, settings (level, contrast, and window), and postprocessing experience used to create the 3D images must be considered.

Despite its accuracy and utility, this technique is limited in several ways. DSA requires a dedicated angiography suite and an experienced operator [25]. Further, as DSA is relatively invasive, costly, and time-consuming, performance of serial studies is challenging [27]. Each procedure has a reported 0.5–2% risk of iatrogenic stroke or catheter-induced vessel injury [1]. Moreover, DSA utilizes iodinated contrast agents and radiation. Studies must be performed judiciously in patients with known iodinated contrast allergies or renal insufficiency [24]. Finally, angiography provides no information about tissue perfusion or ischemia [16]. Despite its utility in assessing vessel caliber, positive findings do not universally correlate with clinical symptomatology.

## 4. Computed Tomographic Angiography (CTA)

**Figure 4:**
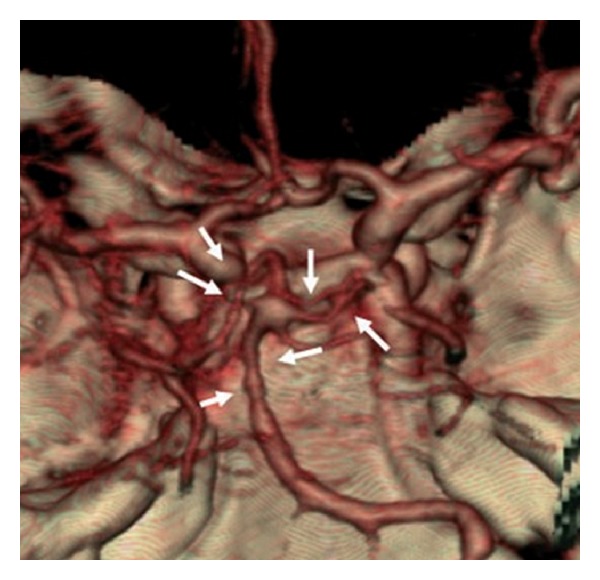
CT Angiography. Three-dimensional reconstruction shows severe vasospasm of the distal basilar artery and bilateral PCAs adapted from [1].

Simultaneous image acquisition with helical CT scanners forms the foundation of CT Angiography (CTA). Since its introduction into clinical practice, technological advances have led to faster imaging with improved spatial resolution. Dynamic scanning has reduced section thickness to submillimeter levels rendering three-dimensional imaging feasible and significantly condensing acquisition times [3]. CTA was first used in the assessment of cerebral vasospasm in 1997 [28]. With the advent of multidetector CTA, the technique requires less contrast medium and optimizes spatial resolution [29] (Figure 4). Modern CTA involves intravenous injection of an iodinated contrast bolus followed by rapid scanning. Acquisition time lasts no more than 1 minute [30–32]. CTA is a fast, reliable, and relatively inexpensive method for detecting decreases in vessel caliber [33–35]. Studies have reported high accuracy in diagnosis of proximal vasospasm, as well as an ability to differentiate between mild, moderate, and severe vessel narrowing [36–38]. Reports of the sensitivity, specificity, and accuracy of CTA as a diagnostic tool have varied considerably among studies with differing patient sample sizes and inclusion criteria. However, a 2010 meta-analysis reported overall sensitivities and specificities of 80%, 93%, respectively [39]. Other advantages include the ability to synchronize the CTA examination with multimodal CT imaging, including noncontrast CT (to assess for hydrocephalus, hemorrhage, completed infarction, etc.) and CT perfusion.

CTA has several limitations. Like many other imaging modalities it is a technique dependent. Proper execution requires correct timing of bolus injection and image acquisition. CTA, especially when performed serially, poses risks of radiation and iodinated contrast exposure. Judgment should be employed in patients with iodinated contrast allergy or impaired renal function. CTA images can be degraded by beam-hardening artifact of nearby clips and coils and often exhibit suboptimal visualization of distal vasculature [1, 29, 30, 38]. Finally, CTA can overrate the degree of vasospasm by underestimating the vessel diameter of large cerebral arteries [40–42]. As CTA is minimally invasive, repeatable, and relatively inexpensive, it has been investigated as a possible surrogate, or substitute, for DSA. Studies have generally shown high correlation between CTA and DSA, especially in cases of more severe vasospasm [29, 36, 40–43]. While CTA remains an adequate method for detecting proximal vasospasm, a negative, or inconclusive, result should not preclude further investigation [38, 42].

## 5. Magnetic Resonance Angiography (MRA)

Magnetic Resonance Angiography (MRA) was introduced as a noninvasive method for vascular imaging in 1986 [44]. Initially utilized for the diagnosis of intracranial aneurysms and atherosclerotic-related vascular stenosis, the technique was not routinely applied to the diagnosis of vasospasm until 1995 [45]. MRA may now be performed using various flow-independent strategies and is capable of addressing both radiographic vessel narrowing and physiological measurements relevant to vasospasm [1, 46].

**Figure 5:**
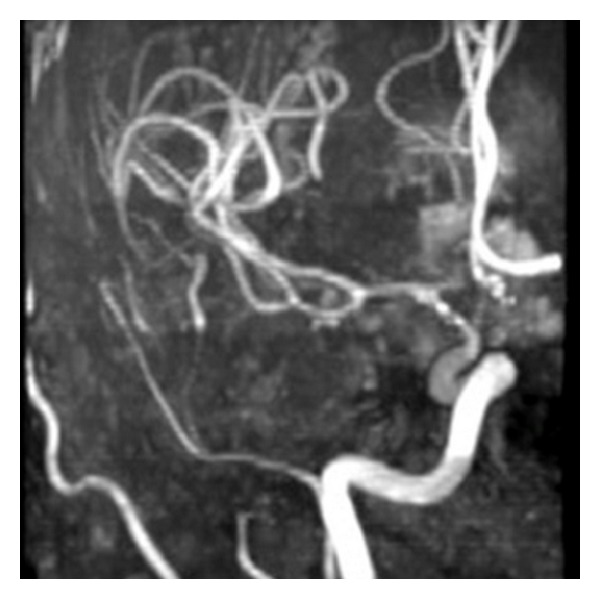
MR Angiography. Vasospasm of the supraclinoid left internal carotid artery, middle cerebral artery, and anterior cerebral artery demonstrated on time of flight MRA.

The standard technique utilized in evaluation of cerebral vasospasm is the time of flight (TOF) sequence. Application centers on the principle that flowing blood experiences less excitation pulse than surrounding static tissue. Blood is excited in the neck by brief pulses and imaged in the brain. As circulating blood remains unsaturated by the excitation pulses, the vessels create higher signal intensity than the surrounding static tissue, allowing for visualization (Figure 5). Studies may be programmed to assess either arterial or venous flow by adjusting the time at which images are captured following excitation pulses [1].

TOF-MRA is noninvasive and requires neither radiation nor contrast exposure [1, 33]. Further, TOF-MRA provides images with relatively high spatial resolution (1 mm) [1, 47] and multiplanar postprocessing capabilities [28]. In an investigation reported in 2000, MRA demonstrated an overall specificity of 97% in demonstrating vasospasm. The technique also exhibited high sensitivity for vasospasm in the ACA (100%). However, it failed to demonstrate high sensitivities for vasospasm in the ICA (25%) and MCA (56%) [46]. It was concluded that while MRA can adequately detect cerebral vasospasm, it remains inferior to DSA in overall accuracy, especially in the ICA and MCA vessels [46, 47]. Owing to a high negative predictive value, TOF-MRA may be useful as a noninvasive screening tool assessing for alternate etiologies of neurological decline [27, 48]. TOF-MRA produces fewer artifacts from platinum embolization coils than CTA and is therefore superior to the latter for imaging the vasculature adjacent to them. However, visualization adjacent to nitinol stents is limited.

TOF-MRA studies require transport to an MRI suite, fairly extensive study times, and prolonged patient cooperation [1, 27]. As the data acquisition sequence is heavily T1 weighted, image artifacts can arise secondary to technical issues. Artifact can be generated by aneurysm clips or residual air from recent surgery, interference due to the presence of methemoglobin in the subarachnoid space, high fat concentrations, or tissue with a naturally high T1 signal [1]. Finally, technical image acquisition issues can result in a limited ability to detect slow flow. Vessel narrowing may be overestimated in the presence of turbulent flow, which creates signal dissipation [48, 49].

An alternate MRA technique is phase contrasted MRA (PC-MRA). This method involves overlaying sequential images and subsequently extracting the signal generated by flowing blood. Assessment can be tailored to specific velocities and directional flow evaluated. While this method allows for higher contrast images and limits T1 artifact, it requires longer acquisition times and is less reliable in assessing for intracranial vessel narrowing. Flow velocities can be measured, but are susceptible to signal loss from turbulence [47–49]. While MRA has advantages over other imaging techniques, it has not been universally accepted as a self-sufficient and reliable diagnostic tool for vasospasm detection and is often employed in conjunction with other studies. 

## 6. Positron Emission Tomography (PET)

**Figure 6:**
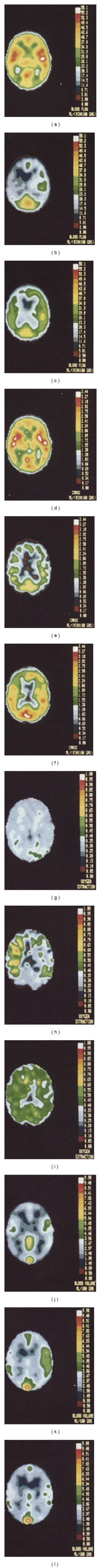
PET. Sequential PET studies of a patient with a ruptured PCA aneurysm. The first study (left column) shows day 2 after rupture with no rCBF abnormalities. The second study (middle column) shows day 10 after rupture with globally decreased rCBF indicating widespread vasospasm in bilateral ICA, MCA, and ACA territories. The third study (right column) shows day 23 after rupture with improved rCBF and relief of vasospasm. The figure is adapted from [51].

Positron Emission Tomography (PET) was initially performed to investigate vasospasm in 1977 [50], but has been sparsely utilized for this purpose over the past 35 years. ^15^O PET is a nuclear imaging technique that involves the injection or inhalation of a radiolabeled ^15^O isotope. This isotope then forms either H_2_^15^O or C^15^O. The radionuclide decays and emits positrons, which collide with an electron causing annihilation. This reaction releases two 511 KeV photons in opposite directions, which can subsequently be detected by a PET scanner. Finally, the scans are correlated with CT images to provide anatomical reference [1, 30] (Figure 6). 

^15^O PET is a noninvasive test that can be repeated. The half-life of the isotope is 122.1 seconds, and the acquisition time for an ^15^O PET image is less than 3 minutes [1, 16, 52]. The rapidity of the test minimizes the radiation dose to the patient. Furthermore, ^15^O PET is considered the radiographic gold standard for determining cerebral perfusion parameters, including cerebral blood flow (CBF), cerebral metabolic rate (CMRO_2_), cerebral blood volume (CBV), oxygen extraction factor (OEF), and cerebrovascular mean transit time (t-CBV/CBF) [1, 30]. ^15^O PET has been utilized to establish threshold CBF values that differentiate reversible (12–20 mL/100 g/min) and irreversible ischemia (<12 mL/100 g/min) [51]. Studies have demonstrated that CBF, CMRO2, and CBV values are decreased in the setting of cerebral vasospasm, suggesting dysfunctional vasodilatory compensation in response to ischemia [53]. 

Despite its utility in providing dynamic information about cerebral hemodynamics and metabolism during vasospasm, ^15^O PET has several practical limitations. Logistically, ^15^O PET is a cumbersome examination, as it requires an expensive on-site cyclotron for isotope creation and patient transfer to a PET scanning machine/facility. As H_2_^15^O and C^15^O are nonlipid soluble tracers, recorded CBF values have inherent imprecision. Inaccuracy is most pronounced when CBF values increase >20 mL/100 g/min [1]. Thus, while ^15^O PET is a valuable tool for assessing cerebral perfusion and understanding the hemodynamics of vasospasm, it is less practical for clinical management and is uncommonly utilized in this setting.

## 7. Single Photon Emission Computed Tomography (SPECT)

Single Photon Emission Computed Tomography (SPECT) using  ^99m^Tc-HMPAO was introduced for the measurement of regional cerebral blood flow in 1986 [54]. Soon thereafter, it was utilized as a novel diagnostic tool for detection of regional hypoperfusion secondary to cerebral vasospasm [55].

**Figure 7:**
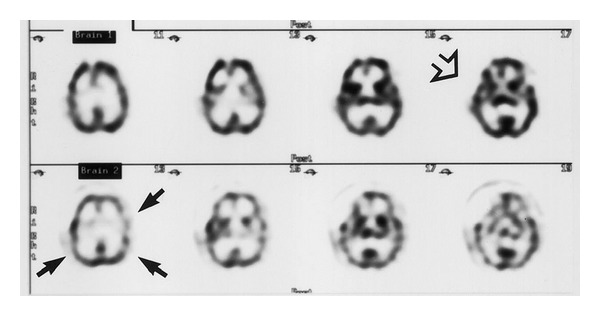
SPECT. Initial transaxial SPECT images (row 1) showing only a postoperative defect from clipping of an ACA aneurysm, demonstrated by the open arrow. Follow-up transaxial SPECT images (row 2) show diffuse hypoperfusion, indicating moderate vasospasm, demonstrated by the closed arrows. The figure is borrowed from [56].

SPECT is a nuclear imaging technique that measures regional cerebral blood flow (rCBF) by assessing flow-dependent uptake of radioactive tracer [56]. The most commonly used tracer is technetium-99 m coupled to a lipophilic compound, hexamethylpropyleneamine oxime ( ^99m^Tc -HMPAO), due to availability and permissive cost. Following intravenous injection, the tracer readily passes the blood brain barrier and is converted to a hydrophilic form that can be retained for long periods of time (half-life of 24–48 hours) [30]. This allows for delayed imaging if necessary. Conversion of the lipophilic radiotracer to a hydrophilic one prevents its redistribution back into the circulation, thus permitting an instantaneous snapshot of cerebral perfusion at the time it is injected intravenously. Isotope tracer concentrations are then measured in 360° rotation using a planar gamma camera, and data is coupled to tomographic sequences to provide anatomical reference [1, 10]. rCBF measurements determined by HMPAO SPECT are defined in a relative (rather than absolute) fashion and are determined by assessing the difference in proportional compound uptake between a region of interest (vasospasm) and an arbitrarily selected “normal” region, usually the cerebellum or contralateral hemisphere [1, 10, 57] (Figure 7). 

This technique is noninvasive, inexpensive, and accessible. Radiopharmaceutical preparation of HMPAO is relatively straightforward, and the technetium gamma emitter is widely available in clinical practice [58]. Studies have repeatedly demonstrated utility in the prediction and detection of delayed ischemic neurological deficit (DIND) in regions of cerebral vasospasm [10, 16, 57, 59] and watershed territories [60]. HMPAO SPECT can reliably detect early changes in relative tissue perfusion and, thereby, assist in differentiating vasospasm-induced hypoperfusion from other causes of neurologic decline [10]. A 1998 study reported the sensitivity and specificity of HMPAO SPECT for detection of vasospasm to be 89% and 75%, respectively. The investigators suggested that a negative HMPAO SPECT test might obviate the need for DSA [61]. HMPAO SPECT has also been utilized to monitor the success of interventional angioplasty in improving regional CBF [57].

Despite these results, the clinical utility of HMPAO SPECT is controversial [27]. On a practical level, the injected compound is difficult to be produced in an emergency setting. Thus, HMPAO SPECT may not be useful as a first-line technique in the setting of potential vasospasm. Further, the images are relatively low in resolution with minimal anatomic detail and granularity, often necessitating and coupling with other imaging modalities [10]. HMPAO SPECT requires radiation, long acquisition times and cannot be rapidly repeated [52]. However, given the relative nature of the CBF measurements provided by HMPAO SPECT, serial studies may actually be necessary to evaluate trends in cerebral perfusion [62]. Finally, the interpretation of the semiquantitative data provided by HMPAO SPECT is based on the assumption that the “affected” regions are being compared to “normal” regions of the brain. This may be an invalid assumption in the setting of diffuse vasospasm, and may affect the interpretation of results. If the patient has symmetric or global perfusion deficit secondary to chronic cerebrovascular disease, postsurgical edema, or peripheral vasospasm then the calculated rCBF values will be inaccurate [1, 10, 56, 63] and may show poor correlation with alternate quantitative cerebral perfusion measurement techniques [64]. 

## 8. ^133^Xenon Clearance Technique

**Figure 8:**
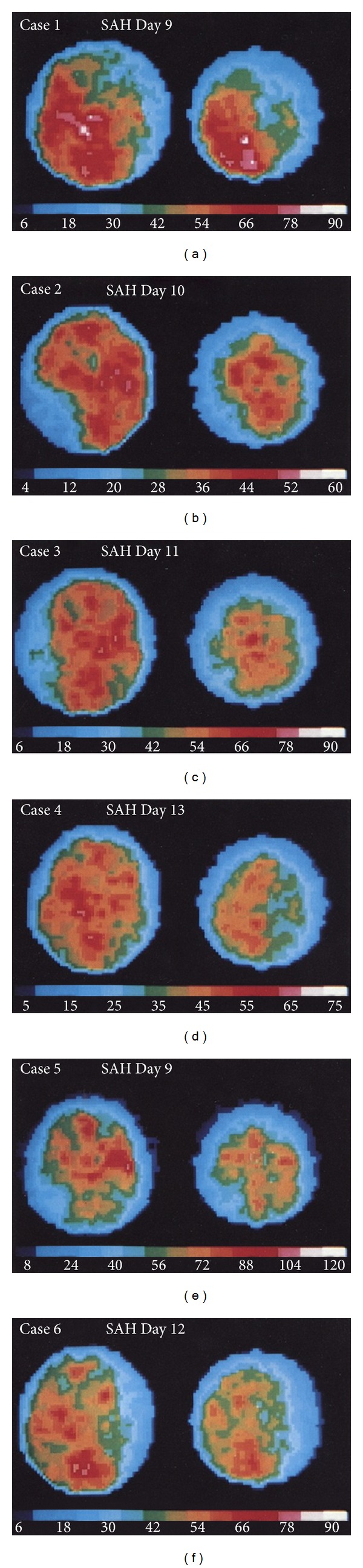
^133^Xenon Clearance. Scans show rCBF pattern defects in 6 patients with cerebral vasospasm and associated DIND. The figure is adapted from [69].

The ^133^Xenon Clearance Technique (^133^Xe) was originally introduced for the purpose of studying regional cerebral blood flow in 1966 [65]. It has been utilized, infrequently, to demonstrate regional hypoperfusion (decreased CBF values) in the setting of vasospasm [52, 66–69]. The technique is a form of emission CT involving either inhalation or intravenous administration of radioactive xenon (^133^Xe) [69]. Xenon, a radio-opaque gas that readily diffuses into brain tissue, is used in various imaging modalities, including ^133^Xenon Clearance and Xenon-Enhanced CT. Bedside collimators detect and quantify ^133^Xe “washout,” and quantitative cerebral blood flow calculations can subsequently be derived from these values [30] (Figure 8). 

This study is minimally invasive and permits delineation of severity and anatomic location of cerebral hypoperfusion [16, 69]. It possesses potential advantages over less accessible and more invasive radiotracer methods. In addition, it is a relatively low cost procedure, and only small doses of radioactive Xenon isotope are required [30, 52]. ^133^Xe clearance has been effectively used to correlate decreased rCBF values with clinical neurological grade (DIND) [67, 70] and provide stable quantitative CBF measurements that do not rely on comparative values [16].

Xenon Clearance provides images with relatively poor spatial resolution. Further, it can overestimate CBF in regions of low flow or stasis due to surrounding isotope uptake, a phenomenon commonly referred to as “look through” [30]. Xenon clearance has not sustained a prominent role in the current diagnosis and management of cerebral vasospasm. 

## 9. Xenon-Enhanced Computed Tomography (Xe/CT)

Xenon-Enhanced Computed Tomography (Xe 133/CT) was first utilized for the analysis of cerebral perfusion in 1978 [71]. The highly diffusible Xenon gas is administered to evaluate intracranial blood flow using a CT scanner. A 72% oxygen 28% stable Xenon mixture is inhaled by the patient over a period of slightly more than 4 minutes, and the brain is subsequently imaged. End tidal CO_2_ and Xenon levels are continuously monitored [1, 10, 52, 72].

Quantitative CBF values are calculated using a modified Key-Schmidt equation (shown below), which factors arterial concentration measurements (measured as end tidal Xenon concentration (Cxe_Art_(*u*)) and tissue arrival times (dependent on blood-brain partition coefficient, **λ**) to assess Xenon concentration changes over time (Cxe_br_(*t*)) [1, 73]:

(2) Cxebr(t)=λK0∫CxeArtt(u)e−k(t−u)du.

**Figure 9:**
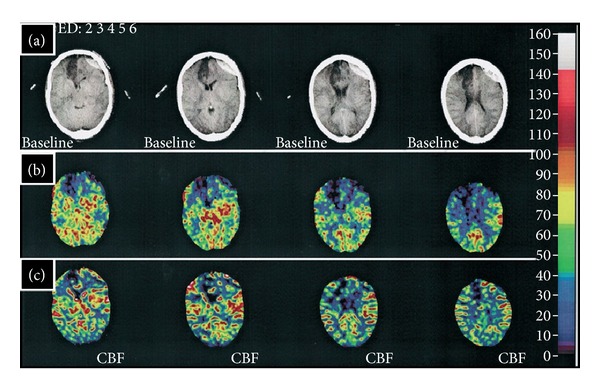
Xenon-Enhanced CT. Baseline CT images (row (a)) indicate a right ACA infarct following aneurysmal rupture of the right ACA. A first Xe/CT study performed with induced hypertension (row (b)) demonstrated a region of ischemia (reduced CBF) in the left frontal region consistent with vasospasm. A second Xe/CT study performed after the withdrawal of pressors (row (c)) did not demonstrate this same region of ischemia. The figure is adapted from [1].

Flow values are represented with a standardized, color-coded graph [1] (Figure 9). Xe/CT overcomes many of the limitations posed by alternative perfusion techniques as it provides standardized, quantitative CBF data with high resolution and direct anatomical correlation [10, 72–75] Measurements of ischemia correlate with clinically significant DIND [1]. Threshold ischemia CBF values for reversible (~15–20 mL/100 g/min) and irreversible ischemia (<~15 mL/100 g/min) [10, 75, 76] correlate with previous threshold values determined using ^15^O PET [51].

The technique is relatively inexpensive. As the arterial half-life of Xenon is approximately 90 seconds, imaging can be performed repeatedly at the bedside with portable CT scanners during periods of high-risk or potential treatment [1, 10, 52, 77, 78]. Studies have suggested that Xe/CT can identify patients at risk for vasospasm in the setting of normal CT imaging [76]. 

Limitations relate to equipment availability, patient cooperation, potential side effects (including the possibility of increased intracranial pressure), and relatively high radiation exposure (15–20 rads) [10, 16, 52]. 

## 10. Dynamic Perfusion Computed Tomography (PCT)

**Figure 10:**
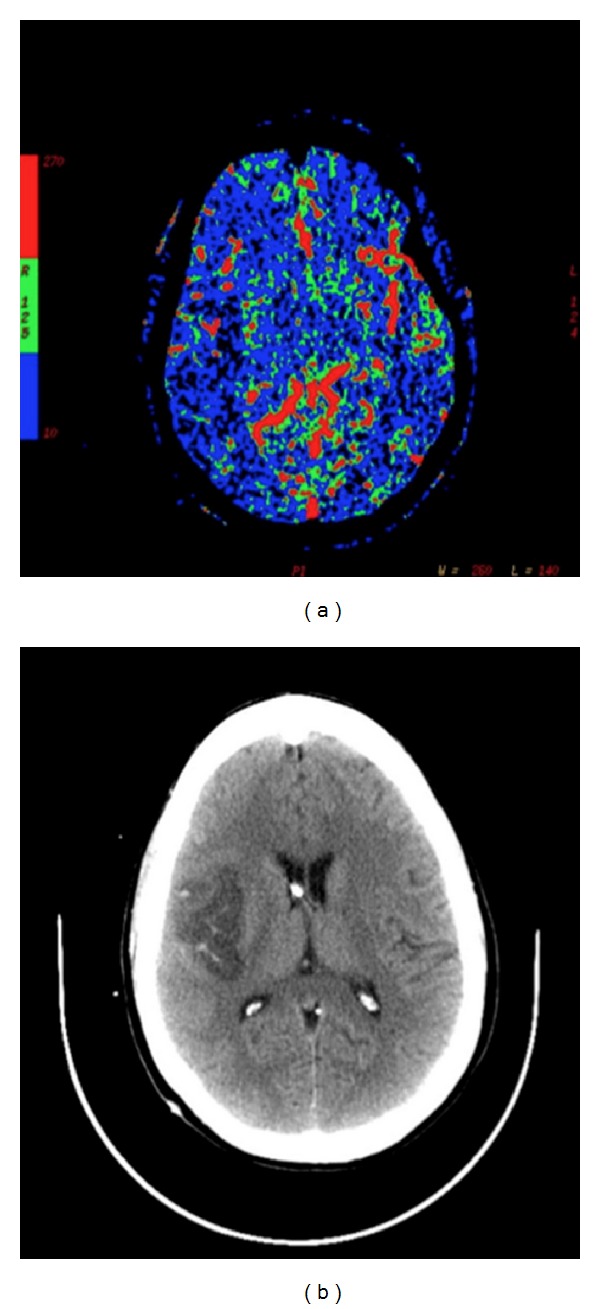
CT Perfusion. Decreased perfusion secondary to vasospasm in the left middle cerebral artery territory on CTP (a). Corresponding ischemia is noted on CT scan (b).

Dynamic Perfusion Computed Tomography (PCT) is an advanced, CT-based technology facilitated by improved scanning speeds and computer applications. Rapid acquisition enables dynamic evaluation of cerebral blood flow [3, 10]. The technique was first applied to cerebral vasospasm in 2000 [79]. PCT utilizes a nondiffusible contrast medium. First-pass concentrations are measured across the intracranial vasculature [80]. Incremental changes in tissue attenuation are measured on serially acquired CT scans and time-density curves constructed [10, 80]. PCT data processing through deconvolution analysis allows for the calculation of cerebral blood flow (CBF), cerebral blood volume (CBV), mean transit time (MTT), and time to peak attenuation. Data can then be used to generate color-coded perfusion maps. The examination is confined to a spatially limited region of interest (ROI), typically at the level of the basal ganglia and lateral ventricles, because this area contains territories supplied by the anterior, middle, and posterior cerebral arteries. CBV and MTT are measured on the time-density curve with CBF calculated according to the central volume principle (Figure 10):

(3) $$\begin{array}{c} \text{MMT}=\frac{\text{CBV}}{\text{CBF}}. \end{array}$$

PCT can generate both qualitative and quantitative analyses of cerebral hemodynamics in a repeatable fashion. It can be performed rapidly and can dynamically monitor the sequelae of vasospasm. A traditional CTA can be obtained simultaneously. The study only requires a helical CT scanner and the software necessary for postprocessing. PCT is able to assess relative perfusion deficits secondary to vasospasm [37, 38]. Recent studies have demonstrated that MTT threshold values can be effectively used to differentiate symptomatic vasospasm from infarction [10, 81, 82]. A 2010 meta-analysis reported sensitivities and specificities of 74.1%, 93%, respectively [39]. PCT has been utilized to assess efficacy of new therapies aimed at reversing hemodynamic dysfunction [34, 35, 83].

PCT has several disadvantages. First, despite rapid advances and technological refinements, the radiation dose remains relatively high. Iodinated contrast should be used judiciously in patients with contrast allergies or impaired renal function [10, 34, 38]. In most facilities, limited anatomical coverage capacities (4-5 axial slices in the ROI) can compromise diagnostic sensitivity [10, 84]. PCT exhibits low accuracy in the posterior fossa [85]. Due to the inherent assumptions of the mathematical model, two parameters are critical to the accuracy of PCT testing: (1) adequate delivery of the contrast to the intracranial vasculature and (2) containment of the contrast within the vasculature. For this reason, patients with marked cardiovascular disease or blood brain barrier disruption are not ideal candidates [80].

## 11. Diffusion-Weighted and MR Perfusion Imaging (DWI/PWI)

Clinically, Magnetic Resonance Imaging (MRI) was not performed until 1981. Techniques and equipment have advanced rapidly since. MR studies now exhibit high-spatial resolution and include three-dimensional capabilities [3]. Diffusion-Weighted (DWI) and MR Perfusion Imaging sequences are tailored protocols, first used to evaluate acute stroke in the 1990s [86]. DWI and MR perfusion imaging are mechanistically different modalities, but are often considered and performed together [10]. Diffusion-Weighted MRI is based on proton motion and diffusion of water molecules. In early phases of permanent ischemic brain injury, water diffusivity is markedly decreased due to failure of the Na,K-ATPase pump in cellular membranes, which produces increased signal on DWI images [16, 87]. Perfusion MR imaging is similar to Perfusion CT in that it evaluates cerebral hemodynamics. There are two perfusion applications: arterial spin labeling (ASL) and dynamic susceptibility contrast imaging (DSCI). ASL is utilized more frequently for experimental purposes, while DSCI is employed clinically. DSCI involves the injection of gadolinium (a paramagnetic contrast agent) and measurement of concentration changes related to the first-pass bolus. T2 relaxation changes are proportional to the degree of perfusion [1, 10].

**Figure 11:**
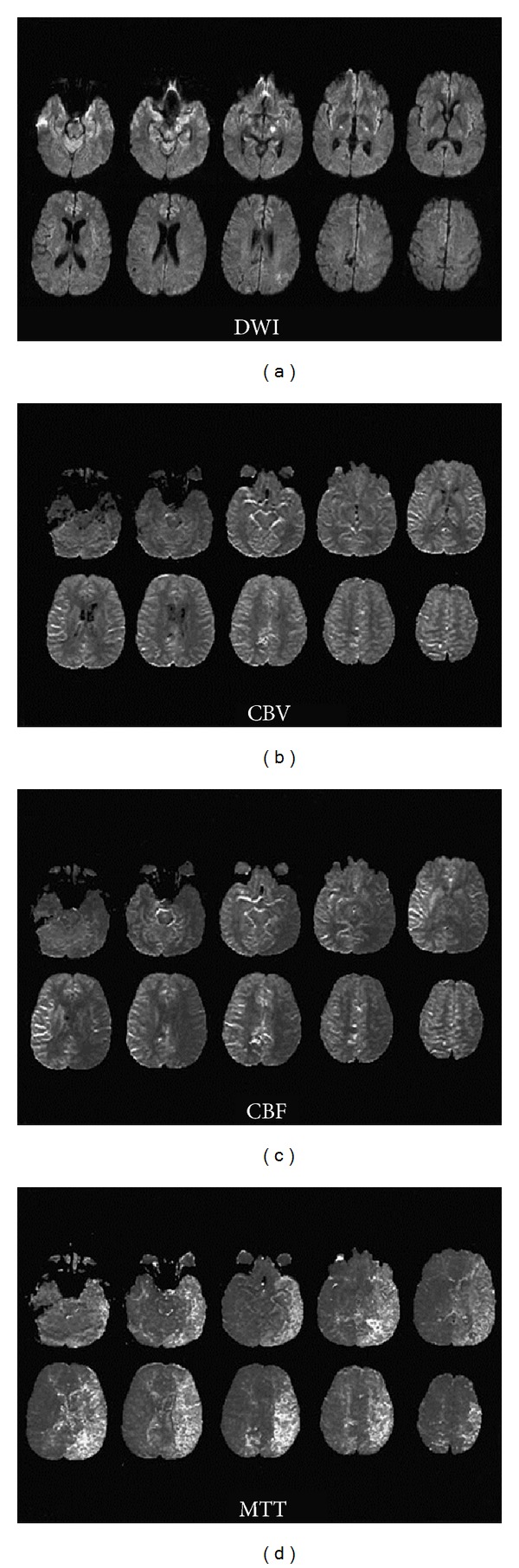
DWI and MR Perfusion. A patient with severe left-sided and moderate right-sided vasospasm demonstrates diffuse hyperintense signal in the posterior parietal lobe as well as a midbrain lesion on DWI (a), while MR Perfusion shows normal rCBV (b), low rCBF (c), and increased tMTT (d) in the left MCA region. This clearly demonstrates the diffusion-perfusion mismatch phenomenon. Originally published in [16].

Unlike Perfusion CT, Diffusion/Perfusion MR techniques allow whole brain imaging and evaluation of cerebral perfusion deficits without radiation exposure [1, 10, 35, 88]. DWI does not require contrast [1] and is capable of providing information on tissue viability in the acute phase. It is sensitive (88–100%) in detecting early brain infarction and assessing cytotoxic tissue edema [1, 10, 16, 89, 90]. Apparent diffusion coefficient (ADC) values are used to confirm true diffusion restriction on DWI sequences. [89]. Perfusion imaging can be utilized to evaluate for cerebral blood flow parameters and extrapolate autoregulation capacity. CBF, CBV, mean time to enhance, and time to peak values are measured and displayed graphically [1, 16, 91]. Coupling of DWI and MR perfusion imaging can reveal a potential “mismatch,” differentiating salvageable ischemic penumbra from infarcted core [10, 16, 27, 91–94] (Figure 11). Delineation of the ischemic penumbra may help guide therapeutic and interventional management decisions. Advanced perfusion-based MR imaging modalities, such as permeability sequences, may also hold promise in early diagnosis of cerebral vasospasm. Detection of blood brain barrier breakdown might suggest “at risk” tissue prior to vessel caliber changes or frank perfusion deficits.

DWI and perfusion imaging remain limited by cost, availability, and time requirements. These methods require patient transportation and cooperation and remain susceptible to artifacts from methemoglobin and certain aneurysm clips [1, 10, 27, 35]. Tissue anisotropy presents an inherent problem, causing potential variability in ADC values [1, 87, 89]. Further, universally available perfusion applications generate only relative rather than absolute flow values. Consensus has not been reached as to which parameters most accurately define the threshold of ischemic core and penumbra [1, 10]. 

## 12. Conclusion 

Multiple advanced imaging modalities have been applied to the diagnosis of cerebral vasospasm. Each has advantages and inherent limitations. As technology and conceptual models continue to progress, more relevant data will be generated and understanding acquired. The synthesis of techniques that assess both cerebral vasculature and brain tissue function may provide meaningful correlation of structural and physiologic imaging parameters with clinical findings.
